# The critical role of the C-terminal lobe of calmodulin in activating eukaryotic elongation factor 2 kinase

**DOI:** 10.1016/j.jbc.2025.110650

**Published:** 2025-08-28

**Authors:** Kimberly J. Long, Luke S. Browning, Andrea Piserchio, Eta A. Isiorho, Mohamed I. Gadallah, Jomai Douangvilay, Elizabeth Y. Wang, Justin K. Kalugin, Jennifer S. Brodbelt, Ranajeet Ghose, Kevin N. Dalby

**Affiliations:** 1 Division of Chemical Biology and Medicinal Chemistry, The University of Texas, Austin, Texas, USA; 2 Interdisciplinary Life Sciences Graduate Program, The University of Texas, Austin, Texas, USA; 3 Department of Chemistry and Biochemistry, The City College of New York, New York, New York, USA; 4 Macromolecular Crystallization Facility CUNY Advanced Science Research Center, New York, New York, USA; 5 Pharmaceutical Analytical Chemistry Department, Faculty of Pharmacy, Assiut University, Assiut, Egypt; 6 College of Natural Sciences, The University of Texas, Austin, Texas, USA; 7 PhD Program in Biochemistry, The Graduate Center of CUNY, New York, New York, USA; 8 PhD Program in Chemistry, The Graduate Center of CUNY, New York, New York, USA; 9 PhD Program in Physics, The Graduate Center of CUNY, New York, New York, USA

**Keywords:** calmodulin (CaM), calcium regulation, elongation factor 2 kinase (eEF-2K), protein chimera, structure-function, calmodulin-dependent activity

## Abstract

Eukaryotic elongation factor 2 kinase (eEF-2K), a member of the α-kinase family, modulates translational rates by phosphorylating eEF-2, a GTPase that facilitates the translocation of the nascent chain on the ribosome during the elongation phase of protein synthesis. eEF-2K is regulated by diverse cellular cues, many of which sensitize it to the Ca^2+^-effector protein calmodulin (CaM). CaM, which binds and allosterically activates eEF-2K in the presence of Ca^2+^, contains two structural “lobes,” each with a pair of Ca^2+^-binding EF hands. Using kinetic analysis, we demonstrate that the isolated C-terminal lobe of CaM (CaM_C_) is sufficient to engage and fully activate eEF-2K in a Ca^2+^-dependent fashion. Genetically fusing CaM_C_ to the N terminus of eEF-2K, upstream of its critical CaM-targeting motif *via* a flexible 2-glycine linker, results in a chimeric species (CaM_C_ is linked to N-truncated eEF-2K [C-LiNK]) that is constitutively active independent of external CaM and Ca^2+^. A structure of the C-LiNK functional core reveals no substantial deviation in the overall conformations of the structural modules and orientations of key catalytic-site residues relative to the heterodimeric complex between full-length CaM and eEF-2K. These observations demonstrate that, in contrast to other CaM-regulated kinases, CaM_C_ alone is sufficient to activate eEF-2K fully. The proximity effect of CaM_C_ in the context of C-LiNK removes the requirement for external Ca^2+^, whose apparent role is to enhance the CaM affinity of eEF-2K and drive kinase activation. Further, the responsiveness of eEF-2K to regulatory stimuli in cells appears to be lost in C-LiNK, presumably due to its permanently “on” state.

In eukaryotic cells, the GTPase eukaryotic elongation factor 2 (eEF-2) promotes the GTP-dependent translocation of the nascent chain from the ribosomal amino-acyl site to the peptidyl site during translational elongation. The ability of eEF-2 to associate with the ribosome is regulated by phosphorylation at Thr-56, which is mediated by the atypical serine/threonine kinase, eukaryotic elongation factor 2 kinase (eEF-2K) (1, 2, 3, 4, 5), a member of the α-kinase family (6, 7). The activity of eEF-2K is finely tuned to cellular energy levels, nutrient availability, and environmental stress, allowing it to dynamically regulate protein synthesis in response to specific cellular cues (8, 9, 10). It has been demonstrated that fundamental processes, such as memory formation and stress adaptation, rely on changes in elongation rates, which play a crucial role in translational reprogramming (11, 12). This adaptive process prioritizes the translation of specific mRNAs by modulating the corresponding rates relative to global translation. Given its pivotal role in regulating protein synthesis, it is not surprising that dysregulated eEF-2K activity correlates with various diseases, including Alzheimer's-related dementia (13), Parkinson’s disease (14), and tumorigenesis (15, 16).

eEF-2K is activated by the Ca^2+^-sensor calmodulin (CaM) (2, 17) *via* an unconventional allosteric mechanism (18, 19) that sets it apart from most CaM-regulated kinases, which are typically activated through CaM-induced displacement of an autoinhibitory segment (20, 21). However, recent studies suggest that alternative modes of CaM-mediated regulation may also occur in other kinases, such as protein serine kinase H1 (22) and CHK2 (23), both of which are members of the CaMK group.

Following CaM binding, eEF-2K undergoes rapid autophosphorylation at an activating site (T348, Fig. 1) within its regulatory loop (R-loop, Fig. S1). The engagement of phosphorylated T348 (*p*T348) at a basic phosphate-binding pocket yields a conformational state capable of efficiently phosphorylating eEF-2. Recent crystal structures of the complex between CaM and a minimal CaM-activatable core of eEF-2K (eEF-2K_TR_, Fig. S1) phosphorylated at T348 (*p*eEF-2K_TR_) provide valuable structural insight into the activation mechanism of eEF-2K (18, 24, 25). The structures suggest intimate interactions between the Ca^2+^-loaded C-terminal lobe of CaM (CaM_C_) and eEF-2K; the N-terminal lobe of CaM (CaM_N_) does not appear to make any stable contacts within the complex. This mode of interaction is consistent with changes in the degree of protection observed for CaM_N_ and CaM_C_ upon formation of the CaM•*p*eEF-2K_TR_ complex, as determined by hydrogen/deuterium exchange mass spectrometry analyses (26).

**Figure 1:**
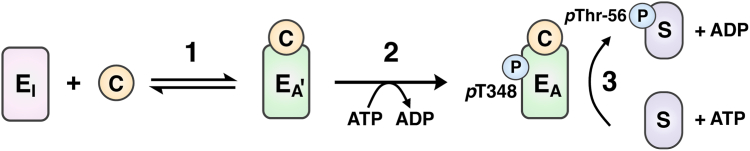
**Schematic representation of the CaM-mediated activation of eEF-2K.** CaM (*yellow circle*) activates eEF-2K through a two-step process. In the first step (1), CaM binds the eEF-2K that is in the inactive state (E_I_), leading to a state (E_A’_) that has high activity toward T348. In the second step (2), rapid autophosphorylation of T348 and its subsequent engagement in a phosphate-binding pocket lead to a fully activated state (E_A_) that can phosphorylate the substrate eEF-2 (S) on Thr-56 with high efficiency. CaM, calmodulin; eEF-2K, eukaryotic elongation factor 2 kinase.

While structural data highlight the importance of CaM_C_ in the eEF-2K complex, a thorough biochemical assessment of the lobe-specific role of CaM in driving eEF-2K activation and activity is needed in the context of the intact enzyme *in vitro* and in cells. For instance, the absence of a substantial fraction of the dynamic R-loop in the eEF-2K_TR_ construct (Fig. S1) may result in the loss of specific regulatory interactions, potentially with CaM_N_. To investigate the contributions of the individual lobes of CaM in eEF-2K function, we assessed the ability of individual constructs encoding CaM_N_ or CaM_C_ to engage and activate eEF-2K in a Ca^2+^-dependent fashion. We found that while the isolated CaM_N_ does not significantly stimulate eEF-2K activity, CaM_C_ displays a robust ability to bind and activate the enzyme in a Ca^2+^-dependent manner. By tethering CaM_C_ to the N terminus of eEF-2K, we generated a constitutively active chimeric species whose activity toward exogenous substrate is insensitive to external Ca^2+^. Additionally, this chimeric construct expressed in MCF10A *eef2k*^*−/−*^ cells displayed high activity even in the presence of typically suppressive posttranslational modifications (PTMs). These findings support a model wherein CaM, specifically CaM_C_, acts as an essential allosteric activator of eEF-2K, with regulatory signals, including Ca^2+^ and diverse PTMs, dynamically modulating its interaction with eEF-2K to regulate its activity.

## Results

### CaM_C_ fully activates eEF-2K

**Figure 2:**
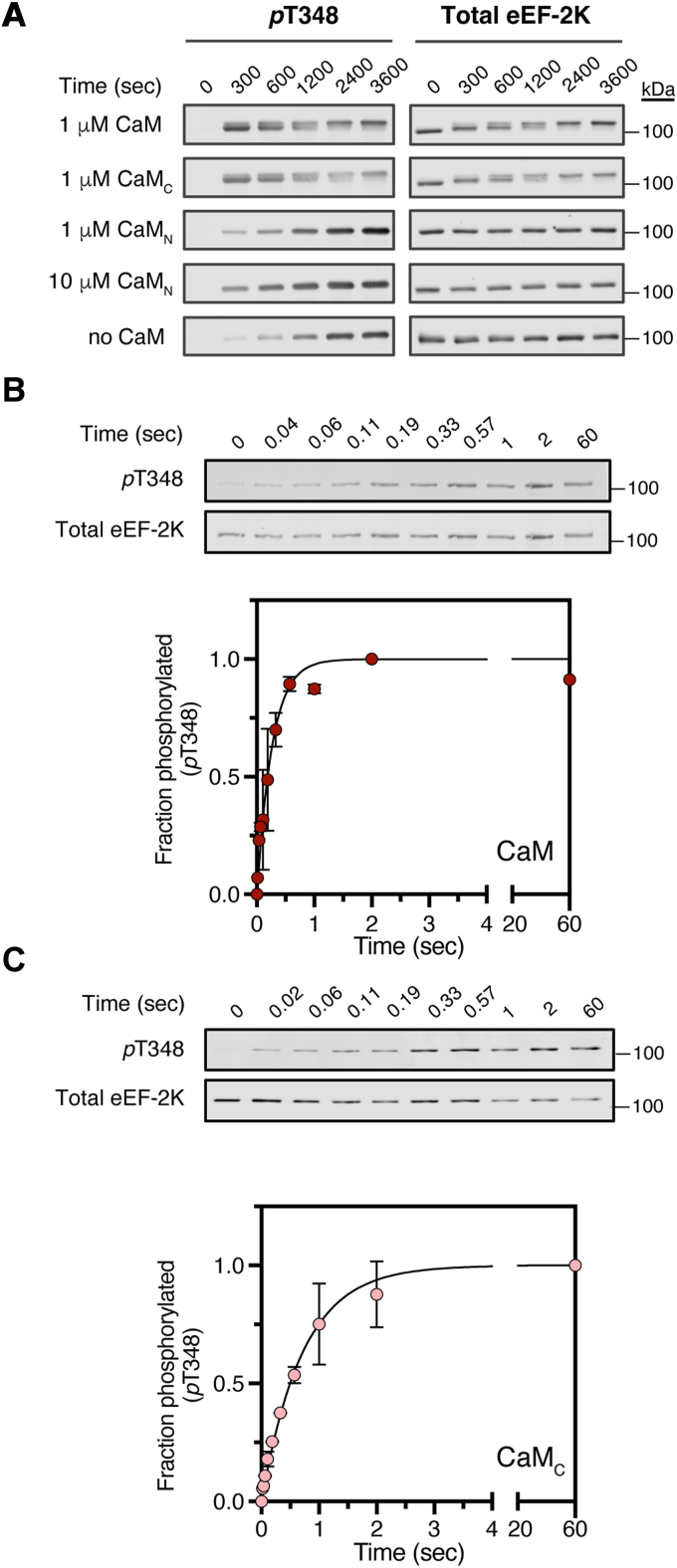
**CaM_C_ stimulates eEF-2K autophosphorylation.***A*, eEF-2K (300 nM) activity toward the primary autophosphorylation site T348 was measured with 1 μM CaM, 1 μM CaM_C_, 1 or 10 μM CaM_N_ or no CaM, in 50 μM free Ca^2+^. Reactions were initiated with 1 mM Mg^2+^•ATP and quenched at various times by addition of hot SDS-loading buffer. Western blot detected *p*T348 and total eEF-2K. Representative data from two independent experiments are shown. *B*-*C*, rapid quench-flow analysis measured T348 autophosphorylation rates. eEF-2K (200 nM) was preincubated with 2 μM CaM (*B*) or 2 μM CaM_C_ (*C*) at 50 μM free Ca^2+^, rapidly mixed with 1 mM Mg^2+^•ATP and quenched at different times. Western blots quantified phosphorylation as fraction of maximal control values (2 or 60 s for CaM or CaM_C_, respectively). Data (mean ± SD, n = 2) were fit to Equation 1 yielding apparent autophosphorylation rate constants ($k_{\text{auto}}^{\text{app}}$) of 3.8 ± 0.38 s^−1^ for CaM and 1.4 ± 0.09 s^−1^ for CaM_C_. CaM, calmodulin; CaM_C_, C-terminal lobe of CaM; CaM_N_, N-terminal lobe of CaM; eEF-2K, eukaryotic elongation factor 2 kinase.

Given that autophosphorylation at T348 represents a key step in eEF-2K activation, we first examined the ability of the individual CaM lobes to stimulate eEF-2K activity toward this site. We utilized constructs encoding the individual lobes of CaM, as established by Sorensen and Shea (27), denoted CaM_N_ (residues 1–80) or CaM_C_ (residues 76–148). We incubated unphosphorylated eEF-2K (28) with ATP in the presence of 50 μM Ca^2+^ with or without 1 μM full-length CaM (referred to as CaM), CaM_N_, or CaM_C_. We quenched the reactions at various time points over an hour and monitored T348 phosphorylation by Western blotting (Fig. 2*A*). Previous studies have reported that CaM drives rapid T348 autophosphorylation (*t*_1/2_ = 0.33 s) (29). Indeed, the reaction was complete by the first time point, taken at 300 s, in the presence of CaM. Autophosphorylation in the presence of CaM_C_ was similarly complete at the first time point. In contrast, in the presence of 1 or 10 μM CaM_N_, the rate of phosphate incorporation was minimally enhanced (∼4-fold with 10 μM CaM_N_) compared to that in the absence of CaM (Fig. S2). These data indicate that the isolated CaM_N_ cannot efficiently drive the activating autophosphorylation of eEF-2K under saturating Ca^2+^ and physiological concentrations of CaM.

We performed rapid quench measurements to quantify T348 autophosphorylation stimulated by CaM and CaM_C_. eEF-2K was preincubated with 4 μM CaM or CaM_C_ and 50 μM free Ca^2+^ and then rapidly mixed (∼2 ms) with saturating Mg^2+^•ATP. Reactions were quenched at various times and quantified by Western blotting for *p*T348 and total eEF-2K (Fig. 2, *B* and *C*). The apparent first-order rate constants for autophosphorylation ($k_{\text{auto}}^{\text{app}})$, obtained by fits to Equation 1 (see Experimental procedures), were found to be similar for CaM (3.8 ± 0.4 s^−1^) and CaM_C_ (1.4 ± 0.1 s^−1^) (Table 1).

**Table 1:** Rate of T348 autophosphorylation by eEF-2K

CaM construct	[CaM] (μM)	$k_{\text{auto}}^{\text{app}}$ (s^−1^)	Rate enhancement
CaM	2	3.8 ± 0.4	7500
CaM_C_	2	1.4 ± 0.1	2800
CaM_N_	10	0.0020 ± 0.0001	3.9
CaM_N_	1	0.0006 ± 0.00004	1.2
No CaM	0	0.00051 ± 0.00006	1

eEF-2K used in the autophosphorylation assays was coexpressed with λ-PP to yield a form of the enzyme without detectable phosphate at T348. Reaction conditions were as follows: [eEF-2K] = 200 nM, [Ca^2+^]_free_ = 50 μM, [ATP] = 1 mM, and the specified amount of various CaM constructs were used. An aliquot of the reaction was quenched at various time points, and the amount of *p*T348 was determined by Western blotting for *p*T348 and total eEF-2K. Data from n = 2 (or n = 3 for no CaM) independent replicates were fit to Equation 1 to determine the apparent rate of T348 autophosphorylation ($k_{\text{auto}}^{\text{app}}$), reported as the mean ± SD. The rate enhancement is defined as the $k_{\text{auto}}^{\text{app}}$ for each reaction divided by the $k_{\text{auto}}^{\text{app}}$ for no CaM.

λ-PP, lambda protein phosphatase; CaM, calmodulin; eEF-2K, eukaryotic elongation factor 2 kinase.

**Figure 3:**
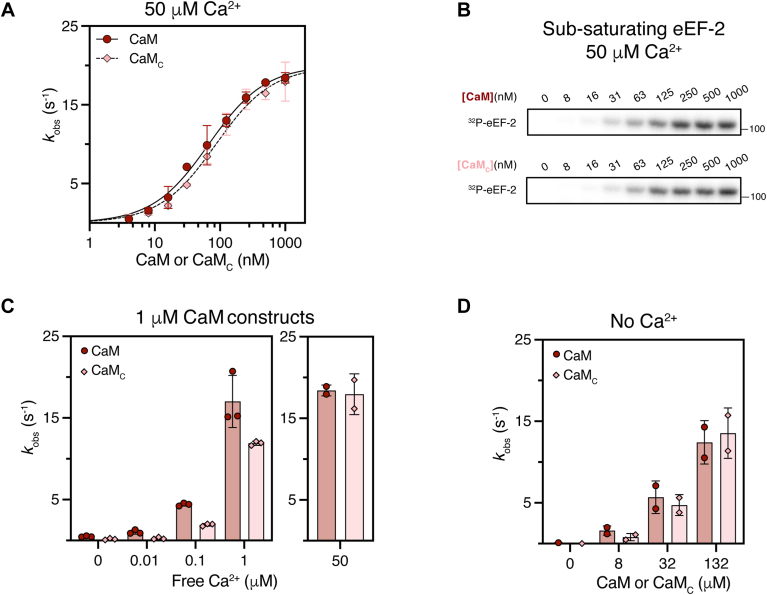
**CaM_C_ binds and activates eEF-2K in a Ca^2+^-sensitive manner.***A*, dose-dependent activation of eEF-2K (1 nM) by CaM or CaM_C_ was measured using 150 μM peptide substrate in 50 μM free Ca^2+^ and 1 mM [γ-^32^P]-ATP. Observed rate constants ($k_{\text{obs}}$, mean ± SD, n = 2) were plotted *versus* CaM concentration and fit to Equation 2 to derive $k_{\text{obs}}^{\max}$ and $K_{\text{CaM}}^{\text{app}}$. Parameters: $k_{\text{obs}}^{\max}$ = 20 ± 0.6 s^−1^ and $K_{\text{CaM}}^{\text{app}}$ = 67 ± 7 nM for CaM; $k_{\text{obs}}^{\max}$ = 20 ± 0.7 s^−1^ and $K_{\text{CaM}}^{\text{app}}$ = 85 ± 10 nM for CaM_C_. *B*, eEF-2K (0.5 nM) activity toward 5 μM yeast eEF-2 was measured with varied CaM or CaM_C_ concentrations in 50 μM free Ca^2+^ and 1 mM [γ-^32^P]-ATP. Reactions were quenched by hot SDS-loading buffer and analyzed by SDS-PAGE. Phosphorimaging visualized ^32^P incorporation (n = 4). Gel bands were quantified by scintillation counting. $k_{\text{obs}}$ values were plotted and fit to Equation 2, yielding $k_{\text{obs}}^{\max}$ = 6.1 ± 1.5 s^−1^ and $K_{\text{CaM}}^{\text{app}}$ = 121 ± 41 nM for CaM; $k_{\text{obs}}^{\max}$ = 5.6 ± 1.4 s^−1^ and $K_{\text{CaM}}^{\text{app}}$ = 156 ± 64 nM for CaM_C_ (Fig. S3). *C*, activity of 2 nM eEF-2K toward 150 μM PepS was measured in the presence of 1 μM CaM or CaM_C_ with varying free Ca^2+^ (0–1000 nM). $k_{\text{obs}}$ values (mean ± SD, n = 3) are shown as bars. Individual data from (*A*) at 50 μM free Ca^2+^ and 1 μM CaM or CaM_C_ are replotted for comparison. *D*, activity of 2 nM eEF-2K with varying CaM or CaM_C_ concentrations in the absence of Ca^2+^ was measured using 150 μM PepS. Corresponding $k_{\text{obs}}$ values are shown (mean ± SD, n = 2). CaM, calmodulin; CaM_C_, C-terminal lobe of CaM; eEF-2K, eukaryotic elongation factor 2 kinase; PepS, peptide substrate.

CaM stimulates eEF-2K’s activity toward substrate in a dose-dependent manner, and the phosphorylation status of T348 kinetically influences substrate phosphorylation. Therefore, to elucidate the effect of CaM_C_ on eEF-2K’s activity toward a substrate, we utilized T348-phosphorylated eEF-2K to measure its steady-state activity against a peptide substrate (PepS) under varying concentrations of CaM or CaM_C_ in the presence of 50 μM free Ca^2+^ (Fig. 3*A*). The corresponding dose response of eEF-2K activity to the concentration of CaM fitted to Equation 2 (see Experimental procedures) yielded similar $K_{\text{CaM}}$ values (the concentration of CaM at half-maximal activity) for CaM_C_ (85 ± 10 nM) and CaM (67 ± 7 nM). Further, the maximal observed rate constant ($k_{\text{obs}}^{\max}$) with saturating CaM_C_ (20.0 ± 0.7 s^−1^) and CaM (20.0 ± 0.6 s^−1^) were similar in the two cases.

To assess whether CaM_C_ has a similar stimulatory effect relative to CaM toward eEF-2, as noted for PepS, we measured the activity of eEF-2K against yeast eEF-2 using varying concentrations of CaM or CaM_C_ in the presence of Ca^2+^ (Fig. 3*B*). Use of a subsaturating concentration of eEF-2 in this assay ensures that a variation in $k_{\text{obs}}^{\max}$ would be detected as a change in catalytic efficiency. We found that the $K_{\text{CaM}}$ and $k_{\text{obs}}^{\max}$ values obtained for CaM_C_ were not substantially different from those for CaM (Fig. S3).

Although our data suggest that under the tested conditions, CaM binding and eEF-2K activation are unaffected by the absence of CaM_N_, it should be noted that kinase activity was measured with a Ca^2+^: Mg^2+^ ratio (1:200) that highly favors Mg^2+^ (similar to their cellular ratio under resting conditions). It is possible that under these conditions, Mg^2+^ binding to the N-lobe of CaM may mask its contributions to complex formation (18, 30). To exclude this scenario, we measured the $K_{\text{CaM}}$ and $k_{\text{obs}}^{\max}$ for eEF-2K against PepS with CaM and CaM_C_ using an altered Ca^2+^/Mg^2+^ ratio (1:10). Under these conditions, the sensitivity of eEF-2K to CaM or CaM_C_ showed no apparent difference (Fig. S4).

Ca^2+^ enhances the $K_{\text{CaM}}$ of eEF-2K toward a PepS by approximately 500-fold (28, 29). To investigate whether a similar effect exists for CaM_C_, we measured eEF-2K activity against PepS with a fixed concentration of CaM or CaM_C_ at various concentrations of free Ca^2+^ (Fig. 3*C*). Previous studies have reported that the Ca^2+^ affinity of the CaM_C_ construct is slightly reduced compared to CaM (27). It was, therefore, not surprising that under low Ca^2+^, the activity of 1 nM eEF-2K with 1 μM CaM_C_ was reduced compared to CaM. However, when stimulated with a high concentration of Ca^2+^ (50 μM), CaM_C_ could activate eEF-2K similarly to CaM (Fig. 3*A*). Overall, the activity of eEF-2K with CaM_C_ or CaM increased with increasing Ca^2+^. Given that Ca^2+^ does not appear to directly simulate eEF-2K in the absence of CaM (28), these data suggest that Ca^2+^ bound to CaM_C_ enhances eEF-2K activity by promoting complex formation. Notably, CaM and CaM_C_ show comparable abilities to activate eEF-2K in the absence of Ca^2+^ (Fig. 3*D*).

### Fusing CaM_C_ to eEF-2K leads to a constitutively active kinase

**Figure 4:**
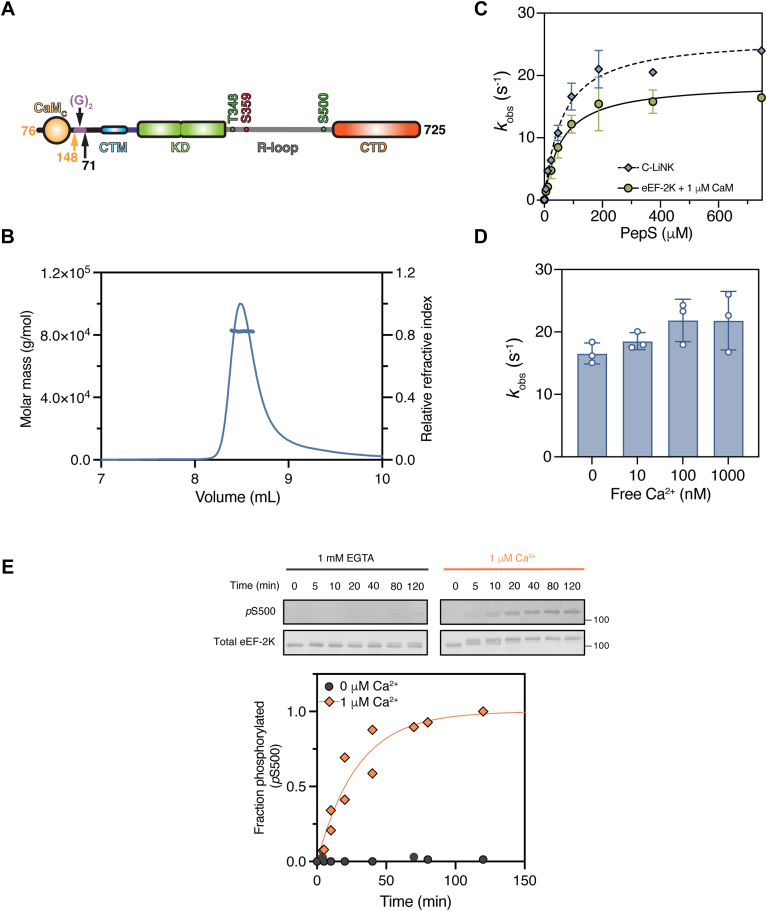
***In vitro* characterization of C-LiNK.***A*, schematic of C-LiNK construct: CaM_C_ (residues 76–148) linked *via* two glycines to N terminally truncated eEF-2K (residues 71–725). Structural elements include CaM-targeting motif (CTM), α-kinase domain (KD), and regulatory loop (R-loop) with activating (T348, S500) and inhibitory (S359) phosphorylation sites. *B*, multiangle light scattering (MALS) of purified C-LiNK shows a monomeric species with molar mass ∼82.4 kDa, consistent with predicted ∼83 kDa. *C*, activity of 1 nM eEF-2K (with 1 μM CaM) or C-LiNK (without added CaM) was measured at varying PepS concentrations with 1 mM [γ-^32^P]-ATP and 50 μM free Ca^2+^. $k_{\text{obs}}$ values (mean ± SD, n = 2) were fit to Equation 3 to yield $k_{\text{cat}}^{\text{app}}$ and $K_{m}^{\text{app}}$: eEF-2K (19 ± 1 s^−1^, 59 ± 12 μM) and C-LiNK (26 ± 1 s^−1^, 61 ± 9 μM). *D*, activity of 1 nM C-LiNK was measured against 150 μM PepS with 1 mM [γ-^32^P]-ATP, 10 mM Mg^2+^, 1 mM EGTA, and varying free Ca^2+^. Independent data points (n = 3) are shown as *open circles*; mean ± SD as bars with *error lines*. *E*, autophosphorylation of C-LiNK (300 nM) at S500 was measured at 30 °C with 0 or 1 μM free Ca^2+^. Reactions were initiated with 1 mM ATP and samples collected over 0 to 120 min. Western blots quantified *p*S500 normalized to total protein; fraction phosphorylated was normalized to 1 μM Ca^2+^ at 120 min. Data were fit to Equation 1. Apparent autophosphorylation rate constant ($k_{\text{auto}}^{\text{app}}$) was 0.00057 ± 0.00006 s^−1^ (t_1/2_ ∼20 min) at 1 μM Ca^2+^; $k_{\text{auto}}^{\text{app}}$ was not determined at 0 μM Ca^2+^. C-LiNK, CaM_C_ is linked to N-truncated eEF-2K; eEF-2K, eukaryotic elongation factor 2 kinase; PepS, peptide substrate.

Based on the evidence above, CaM_C_ can fully activate eEF-2K. The primary role of Ca^2+^ appears to be enhancing the affinity of CaM_C_ for eEF-2K, thereby leading to its activation. Therefore, it can be expected that increasing the local concentration of CaM, specifically of CaM_C_, will enable kinase activation with reduced reliance on stimulatory inputs such as Ca^2+^. Leveraging our previous structural information (18, 24, 25) and our knowledge of eEF-2K activation by CaM_C_ (described above), we developed a chimeric construct (Fig. 4*A*) in which CaM_C_ is linked to N-truncated eEF-2K (C-LiNK). This construct comprises CaM_C_ (76–148) fused to the N terminally truncated eEF-2K (71–725) using a linker consisting of two glycine residues. This linkage places CaM_C_ immediately upstream of the CaM-targeting motif (Fig. S1), which is essential for the CaM/eEF-2K interaction (18, 31). We have previously shown that the disordered N-terminus of eEF-2K is dispensable for its activation by CaM (26). C-LiNK was expressed (Fig. S5, *A* and *B*) and purified similarly to eEF-2K (32), and its monomeric state was confirmed by multiangle light scattering (MALS) that produced a single peak at a molecular mass of ∼82.4 kDa (Fig. 4*B*).

C-LiNK was coexpressed with lambda phosphatase, a procedure used to express eEF-2K devoid of phosphorylation (28). However, despite this procedure, phosphorylation on T348 (using native eEF-2K numbering in all cases) was detected for C-LiNK by Western blot, and further incubation of purified C-LiNK with ATP did not increase the intensity of the *p*T348 signal (Fig. S5*C*).

In addition to T348, eEF-2K can undergo autophosphorylation at several secondary sites. Phosphorylation at many of these sites is highly dependent on CaM (28, 29, 33). Given the apparent high basal activity of C-LiNK, we utilized a bottom-up tandem mass spectrometry (MS/MS) approach to confirm the phosphorylation status of the expressed protein. C-LiNK was proteolytically digested by trypsin and then analyzed using LC-MS/MS. The resulting MS/MS spectra accounted for 99% sequence coverage, and phosphorylation was detected at residues T348 (>99%) and S445 (1%) (Fig. S6). Fragment ion analysis, including assignment of b- and y-ion series with sub-10 ppm mass accuracy, supported high-confidence peptide identification and phosphorylation site assignment (Tables S1 and S2). Next, we confirmed the ability of C-LiNK to autophosphorylate on T348, S445, and S500 by Western blotting using specific antibodies after incubating the purified protein with Mg^2+^•ATP for varying periods. This confirmed that C-LiNK can undergo autophosphorylation at these residues, similar to CaM-stimulated eEF-2K (Fig. S5, *C*–*E*).

To assess C-LiNK activity relative to CaM-stimulated eEF-2K, we compared the enzymatic activities of C-LiNK or eEF-2K (using 1 μM CaM) against PepS in the presence of saturating Ca^2+^. Fits to Equation 3 (see Experimental procedures; Fig. 4*C*) indicate that C-LiNK is fully active against PepS ($k_{\text{cat}}^{\text{app}}$ = 26 ± 1 s^−1^ for C-LiNK *versus* 19 ± 1 s^−1^ for eEF-2K), with similar catalytic efficiency ($k_{\text{cat}}^{\text{app}}$/ $K_{m}^{\text{app}}$ = 0.43 ± 0.05 μM^−1^ s^−1^ for C-LiNK *versus* 0.32 ± 0.05 μM^−1^ s^−1^ for eEF-2K) as CaM-stimulated eEF-2K. The activity of C-LiNK toward PepS was independent of added Ca^2+^ (0–1000 nM of free Ca^2+^; Fig. 4*D*). Extended dialysis against a Ca^2+^-free buffer (1 mM EGTA for 19 h) before measuring the catalytic activity had no impact on the results (Fig. S7).

It has previously been demonstrated that Ca^2+^-CaM is essential for efficient autophosphorylation at the activating S500 site, which also requires prior phosphorylation at T348 (29). To investigate this further using C-LiNK, which is fully phosphorylated at T348, we incubated C-LiNK with Mg^2+^-ATP in the presence of 1 μM free Ca^2+^ and monitored the appearance of *p*S500 over time (Fig. 4*E*). The data revealed that C-LiNK has a similar rate of S500 autophosphorylation (*t*_1/2_ ∼20 min) as eEF-2K in the presence of saturating Ca^2+^-CaM (28). However, this rate is markedly reduced in the presence of 1 mM EGTA without added Ca^2+^ (Fig. 4*E*), suggesting that the autophosphorylation of S500 remains reliant on Ca^2+^ as in the case of eEF-2K.

### Structure of the C-LiNK functional core

**Figure 5:**
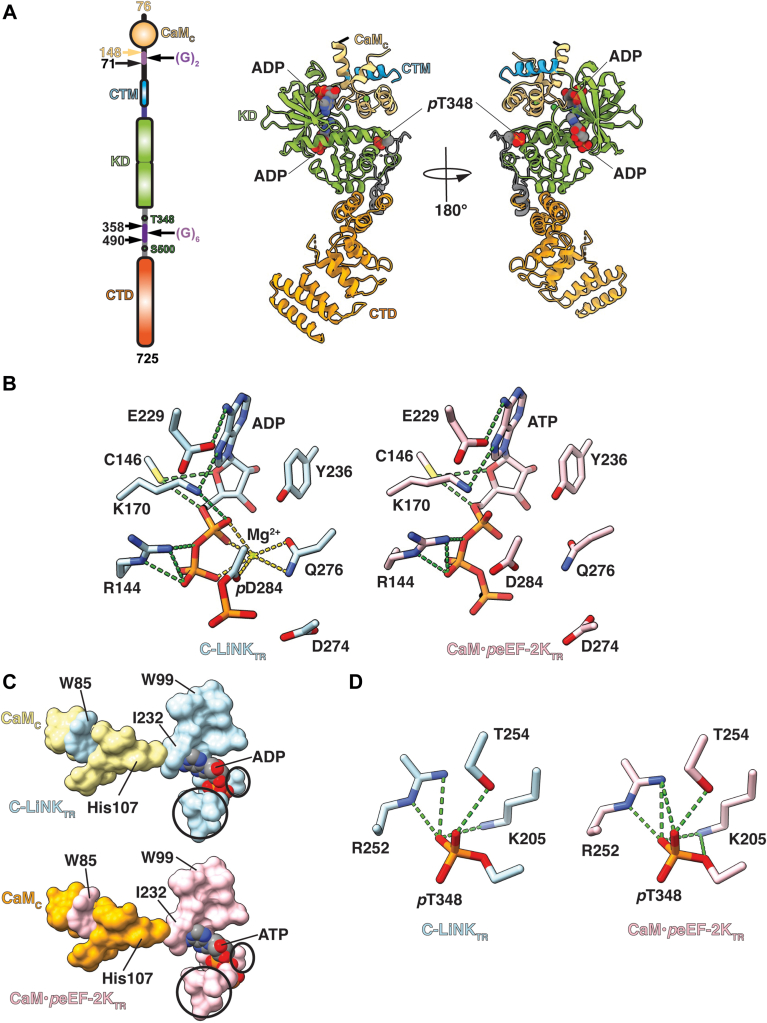
**Structure of C-LiNK_TR_.***A*, the organization of the C-LiNK_TR_ construct is shown schematically on the *left panel*. This construct is derived from C-LiNK (Fig. 4*A*), with an R-loop segment comprising residues 359 to 489 replaced by six glycines, in analogy to eEF-2K_TR_. The structure of C-LiNK_TR_ is shown on the *right panel*, with the key structural modules indicated and colored as on the *left panel*. Two ADP molecules, one bound at the catalytic site and a second bound to the interface between the CaM_C_ module and the N-lobe of the KD, are shown as *spheres*. The phosphorylated T348 (*p*T348) is also indicated. *B*, the conformations of key catalytic site elements in the C-LiNK_TR_ (*left*) and the CaM•*p*eEF-2K_TR_ complex (PDB: 8FNY, *right*) structures show no substantial differences. D284 in the C-LiNK_TR_ structure is phosphorylated and a Mg^2+^ ion is bound at the catalytic site. Hydrogen bonds are indicated by the *green dashed lines* in all cases; the *gold dashed lines* denote heteroatoms with 3.2 Å of the metal center. *C*, the activation spine that links CaM_C_ to the kinase catalytic site through the bound nucleotide is fully formed in C-LiNK_TR_. The geometry of the spine in C-LiNK_TR_ (*top panel*; eEF-2K modules in *light blue*, CaM_C_ in *yellow*) is identical to that seen in the structures of the CaM•eEF-2K_TR_ complex (*bottom panel*; a representative heterodimeric complex, PDB: 8FNY; eEF-2K in *pink*, CaM_C_ in *orange*). Key spine residues are labeled (three-letter codes are used for CaM residues), the nucleotide bound to the catalytic site is shown in both cases, and the active site is *circled*. *D*, the coordination of *p*T348 at the phosphate-binding pocket in C-LiNK_TR_ (*left*) and the 8FNY structure (*right*) is identical. CaM_C_, C-terminal lobe of CaM; C-LiNK, CaM_C_ is linked to N-truncated eEF-2K; eEF-2K, eukaryotic elongation factor 2 kinase; KD, kinase domain; R-loop, regulatory loop.

As noted above, C-LiNK is constitutively active and retains all the properties of eEF-2K upon its activation by CaM (and CaM_C_). One could, therefore, expect that the overall conformation of its functional core, key CaM-recognition modules, and catalytic residues would not be substantially different from that seen in the structures of the CaM•*p*eEF-2K_TR_ complex. To confirm this hypothesis, we determined the structure of C-LiNK_TR_ (Fig. 5*A*) by X-ray crystallography to a resolution of 2.3 Å (see Table 2 for details of data collection, refinement, and structure statistics). The CaM_C_ and eEF-2K structural modules maintain their overall conformations in the context of C-LiNK_TR,_ as seen in the structures of the CaM•*p*eEF-2K_TR_ complex solved in various states (Fig. S8). The average deviation over the CaM_C_ and eEF-2K_TR_ modules was 0.5 ± 0.1 Å (533 ± 12 trimmed residues over five distinct structures). The 7SHQ structure, which shows a greater degree of closure between the kinase domain and C-terminal domain (Fig. S8), was excluded from the average.

**Table 2:** Data collection and structure refinement statistics^a^

Wavelength (Å)	0.92022
Space group	P12_1_1
Unit cell a, b, c (Å)	64.766 59.736 88.766
Unit cell α, β, γ (°)	90.0 98.497 90.0
Resolution range for data processing (Å)	49.39–2.271 (2.352–2.271)
Total number of observations	124,377 (6027)
Total number of unique observations	17,519 (876)
Mean(I)/σ(I)	6.6 (1.6)
Completeness (spherical)	55.9 (9.6)
Completeness (ellipsoidal)^b^	91.7 (63)
Multiplicity	7.1 (6.9)
CC_1/2_	0.994 (0.661)
*R*_merge_ (all I^+^ and I^−^)	0.218 (1.228)
*R*_meas_ (all I^+^ and I^−^)	0.235 (1.328)
*R*_pim_ (all I^+^ and I^−^)	0.088 (0.503)
Reflections used in refinement	17,506 (73)
Reflections used for R_free_	858 (2)
*R*_work_	0.1996 (0.3123)
*R*_free_	0.2559 (0.5003)
Number of non-hydrogen atoms	4636
Macromolecules	4421
Ligands	84
Solvent	155
Protein residues	566
RMS (bonds)	0.011
RMS (angles)	0.71
Ramachandran favored (%)	96.0
Ramachandran allowed (%)	3.64
Ramachandran outliers (%)	0.36
Rotamer outliers (%)	3.04
Clash score	14.65
Average B-factor (Å^2^)	29.32
Macromolecules	29.51
Ligands	32.06
Solvent	22.98
PDB accession code	9OCW

3.411 Å; 0.9481 0.0000 0.3179; 0.970a∗ + 0.244c∗.

2.498 Å; 0.0000 1.0000 0.0000; b∗.

2.253 Å; −0.3179 0.0000 0.9481; −0.229a∗ + 0.973 c∗.

a Statistics for the highest resolution shell are shown in parentheses.

b Diffraction limits and principal axes of ellipsoid fitted to diffraction cut-off surface.

The catalytic site contains a bound ADP molecule and a single Mg^2+^ ion, with the orientations of key side chains of the catalytic residues being essentially unchanged from that seen in the structures of the CaM•*p*eEF-2K_TR_ with a nucleotide (ATP) bound to the catalytic site (Fig. 5*B*). We have previously noted the presence of an “activation spine (A-spine)” that links the CaM-targeting motif engaged CaM_C_ through a conserved regulatory element and the bound nucleotide to the kinase active site (18). The A-spine, although spatially and structurally distinct, bears resemblance to the catalytic spine in conventional kinases, which also utilizes the ATP substrate as a core structural element (34, 35). However, the regulatory (R-) and catalytic spines, characteristic of the active forms of conventional kinases, are absent in α-kinases (18). Not surprisingly, the A-spine is fully formed in C-LiNK, with the constituent residues in similar configurations to the nucleotide-bound form of the CaM•eEF-2K_TR_ complex (Fig. 5*C*).

Surprisingly, even though a fully dephosphorylated C-LiNK was used for crystallization, T348 was found to be phosphorylated and engaged to the phosphate-binding pocket similar to that seen in the structures of the CaM•eEF-2K_TR_ complex (Fig. 5*D*). Additionally, D284, at the catalytic site was also phosphorylated (Fig. 5*B*). Notably, the equivalent residue (D766) in the kinase domain of the homologous *Dictyostelium* myosin heavy chain kinase A has also been seen in the phosphorylated form in multiple crystal structures (36, 37) suggesting that this may represent a common feature in α-kinases. We surmised that an ATP contaminant in the ADP used in the crystallization buffer could be the origin of the phosphates, with the phospho-transfer occurring *in crystallo*. Given that the level of ATP contamination in our ADP stock was ∼2% by NMR analysis, this scenario seems somewhat unlikely.

To test the origin of these multiple phosphorylations despite the apparent lack of ATP, C-LiNK_TR_ was incubated with Ca^2+^ and ADP (from the stock solution used in crystallization) in the absence of Mg^2+^. The resulting molecular mass, measured by electrospray ionization quadrupole time-of-flight mass spectrometry (MS), was consistent with that of the unmodified protein. Adding Mg^2+^ resulted in a shift of ∼80 Da, indicating a single phosphorylation event (Fig. S9) and suggesting that C-LiNK can perhaps utilize ADP to drive phosphotransfer. It has been noted that *Dictyostelium* myosin heavy chain kinase A can also use ADP to drive substrate phosphorylation (36).

### C-LiNK is fully active in cells

**Figure 6:**
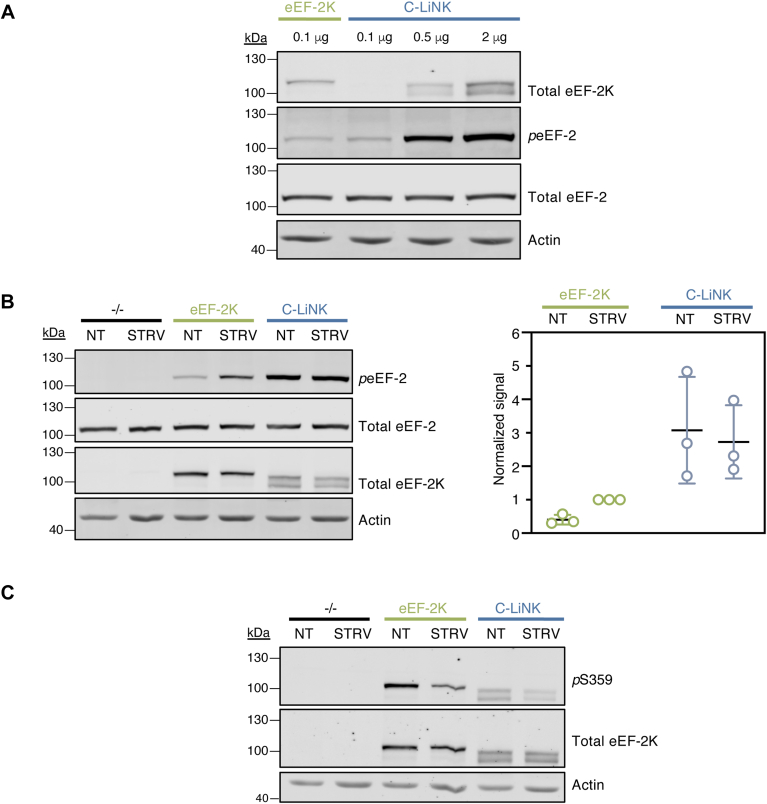
**C-LiNK activity in MCF10A *eef2k*^−/−^ cells is resistant to inhibitory signaling.***A*, MCF10A *eef2k*^−/−^ cells were transfected with indicated amounts of pcDNA3 encoding eEF-2K or C-LiNK. After 16 h, lysates were analyzed by Western blot for eEF-2K, eEF2, phospho-eEF2, and actin (loading control). *B*, cells transfected with empty vector, eEF-2K, or C-LiNK were treated for 2 h with complete media (NT) or starved with DPBS (STRV). A representative Western blot shown on the *left*. The graph (*right*) displays data from (*B*) with the phospho-eEF2 signal normalized to total eEF2 and expressed as a fraction of eEF-2K STRV signal. The mean ± SD (n = 3) is indicated. *C*, cells expressing eEF-2K or C-LiNK were starved or given fresh media; inhibitory phosphorylation at S359 was assessed by Western blot. C-LiNK, CaM_C_ is linked to N-truncated eEF-2K; DPBS, Dulbecco’s PBS; eEF-2, eukaryotic elongation factor 2; eEF-2K, eukaryotic elongation factor 2 kinase.

Our *in vitro* analysis of C-LiNK established that it is fully active and exhibits properties similar to CaM-bound eEF-2K. To assess the cellular properties of C-LiNK, we transfected MCF10A *eef2k*^*−/−*^ cells with pcDNA3 encoding C-LiNK or eEF-2K. Given that previous studies have suggested that high levels of cellular eEF-2K activity correlate with increased degradation (29), it was not surprising that cells displayed lower levels of C-LiNK protein relative to eEF-2K (Fig. 6*A*). Mutation of the D284 (described above) to alanine results in a loss of catalytic activity in eEF-2K (29). Transfection of the D284A mutant of eEF-2K and C-LiNK rescued protein levels, confirming that the low levels of C-LiNK protein (and of eEF-2K) are indeed correlated with its activity (Fig. S10).

To account for the reduced cellular levels of C-LiNK relative to those in eEF-2K, we titrated MCF10A *eef2k*^*−/−*^ cells with increasing amounts of the C-LiNK pcDNA3 vector and immunoblotted for eEF-2K. A 5-fold increase in the concentration of C-LiNK vector relative to eEF-2K vector was sufficient to produce comparable protein levels and was used for further cellular experiments. Interestingly, the eEF-2K antibody recognizes C-LiNK as two distinct bands. A similar banding pattern is observed for eEF-2K *in vitro* when incubated with CaM and ATP (*e.g.,* Figs. 2*A* and 4*E*). We suspect that C-LiNK can undergo hyper-autophosphorylation in cells, which may be the origin of these additional bands. The fact that the kinase-dead (D284A) mutant is recognized as a single band is consistent with this scenario (Fig. S10).

Cellular eEF-2K activity has been reported to be highly sensitive to nutrients. Nutrients and growth factors suppress eEF-2K activity through phosphorylation of residues within the R-loop (*e.g.*, on S359) (38). Removing nutrients (starvation, STRV) from cellular media decreases these inhibitory phosphorylation events and enhances eEF-2 phosphorylation (38). MCF10A *eef2k*^*−/−*^ cells expressing eEF-2K in nutrient-rich (no treatment, NT) media showed lower levels of *p*eEF-2 than cells subjected to STRV (Fig. 6*B*). The activity of C-LiNK, as measured by *p*eEF-2 levels, was higher than eEF-2K in both NT and STRV conditions and displayed no nutrient sensitivity. We also observed a reduction in S359 phosphorylation for both constructs in starved (STRV) MCF10A *eef2k*^*−/−*^ cells as compared to NT cells (Fig. 6*C*). These data indicate that while the levels of inhibitory phosphorylation on C-LiNK can be altered by regulatory inputs, as in eEF-2K, its activity is insensitive to these modifications.

Finally, to confirm that endogenous CaM did not drive C-LiNK activation in the cellular context, we tested the ability of C-LiNK to interact with CaM. We found that CaM labeled with the fluorescent dye N-(iodoacetylaminoethyl)-5-naphthylamine-1-sulfonic acid (IAEDANS) (39) cannot engage C-LiNK, even when present in a 25-fold molar excess. In contrast, eEF-2K shows robust binding to CaM labeled dye (Fig. S11). Thus, it may be concluded that the cellular activity of C-LiNK results from activation in *cis* through its N-terminal CaM_C_ rather than through its interaction with cellular CaM.

## Discussion

CaM’s role as a Ca^2+^ sensor necessitates adaptive conformational changes, which enable it to bind and activate numerous target proteins with varying Ca^2+^ sensitivities (40, 41). Regulation of most CaM targets, including other CaM-regulated kinases, generally requires interaction with both lobes of CaM (42, 43, 44, 45). Although rare, examples in the literature describe targets that can be stimulated to varying degrees by individual lobes of CaM (46). For CaM-regulated kinases, the stimulatory dominance of one lobe of CaM over the other has been noted; for example, the isolated CaM_N_ appears to partially activate CaMKII, whereas CaM_C_ is unable to do so (47). Nevertheless, eEF-2K is distinctive in that maximal activation can be stimulated by the isolated CaM_C_. This lobe also appears to drive the Ca^2+^ sensitivity of activation. CaM lobe-centric activity has been suggested to play a role in the temporal response of decoding diverse Ca^2+^ signals, leading to distinct downstream responses (48, 49). A similar role in interpreting and transducing specific Ca^2+^ fluxes to modulate protein synthesis may also be operative with respect to eEF-2K.

It has been suggested that eEF-2K activity is regulated by Ca^2+^ and PTMs through modulation of its affinity for CaM (8). To investigate this further, we leveraged our structural understanding of the CaM/eEF-2K_TR_ complex and our discovery of CaM_C_'s capacity to activate eEF-2K, creating a constitutively active kinase by genetically linking CaM_C_ to an N terminally truncated eEF-2K (C-LiNK). It is to be noted that this contrasts with most “conventional” CaM-regulated kinases, where deletion of an autoinhibitory or pseudosubstrate segment leads to constitutive activation (50, 51). The Ca^2+^-dependent protein kinases found in plants and protozoans are genetic fusions of CaMK and CaM-like domains. Given that the Ca^2+^-dependent protein kinases are also activated through the “release of inhibition” mechanism operative in conventional CaM-regulated kinases, they may also be constitutively active by deleting the autoinhibitory domain (52, 53, 54). This makes C-LiNK a mechanistically distinct species.

C-LiNK activity toward a PepS appears independent of the presence of Ca^2+^. Indeed, extended incubation with the Ca^2+^-chelator EGTA did not affect the activity. We cannot fully discount the scenario that the fusion greatly enhances the affinity for Ca^2+^ within the fused CaM_C_ to such an extent that the ions cannot be removed through EGTA incubation. Indeed, a marked increase in Ca^2+^ affinity of CaM fused to a CaM-binding peptide has been noted previously (55). Native mass spectrometric analyses proved inconclusive in our case. Nevertheless, the CaM_C_ module within the C-LiNK construct can engage Ca^2+^, as evidenced by the structure of the C-LiNK_TR_ and the fact that Ca^2+^ is necessary to stimulate autophosphorylation at S500 in line with our previous observations for eEF-2K (29). However, the presence of additional Ca^2+^-binding sites elsewhere on eEF-2K, perhaps on the disordered R-loop, that may influence the latter event cannot be ruled out by the present studies.

C-LiNK appears fully active when transiently expressed in MCF10A *eef2K*^−/−^ cells under both nutrient-rich and nutrient-deprived conditions. This contrasts with eEF-2K, whose activity is enhanced by nutrient deprivation. Under nutrient-rich conditions, the inhibitory phosphorylation (S359) status on C-LiNK mimicked that of eEF-2K. However, the activity of C-LiNK, as measured by *p*eEF-2 levels, was sustained despite this suppressive phosphorylation, suggesting that C-LiNK is active under normally inhibitory conditions. This is consistent with the scenario in which inhibitory phosphorylation downregulates eEF-2K activity by reducing CaM binding but has a negligible effect once CaM is stably bound to eEF-2K.

Although the stimulatory contribution of CaM_N_ was minimal under our conditions, it may have an alternative role that has not been explored here. It is possible that CaM_N,_ which is more sensitive to rapid Ca^2+^ transients, could play a role in eEF-2K activity upon induction of specific Ca^2+^ signals (49). It is also possible that CaM_N_ could play a greater role under the influence of specific PTMs not explored in this study. Additional experiments are needed to assess the presence and magnitude of these effects.

## Experimental procedures

### Protein expression and purification

#### CaM constructs

Recombinant CaM_N_ (1–80) was purchased from the University of Iowa Proteomics Facility. Recombinant CaM and CaM_C_ (76–148) were expressed and purified as described previously (47, 56). Briefly, CaM in pET-23 or CaM_C_ in T7-7 (a gift from Dr Madeline Shea, University of Iowa) (27) vectors were expressed in BL21 (DE3) pLysS *Escherichia coli* cells. The cell pellets were resuspended in 50 mM Tris (pH 7.5) containing 10 mM CaCl_2_ and protease inhibitors (TPCK, PMSF, and benzamidine), and then sonicated to lyse the cells. The lysate was centrifuged at 27,200*g* for 30 min at 4 °C, and the supernatant was collected. The CaM constructs were purified by heating the supernatant to 80 °C for 20 min, followed by centrifugation at 2000*g* for 15 min at 22 °C, and then filtering the resulting supernatant. The sample was then applied to a HiPrep Phenyl FF 16/10 column and washed with two column volumes (CVs) of purification buffer A [50 mM Tris (pH 7.5) with 10 mM CaCl_2_] and eluted with 2 CV of purification buffer A containing 0.5 M NaCl or purification buffer B [50 mM Tris (pH 7.5) with 10 mM EGTA]. Then, the sample was passed through a HiPrep 26/10 desalting column (Amersham Biosciences), applied to a Mono-Q HR 10/10 (Amersham Biosciences), washed with 5 CV of 50 mM Tris (pH 7.5), and eluted over 20 CV with a gradient up to 250 mM KCl in 50 mM Tris (pH 7.5). The sample was dialyzed into 25 mM Hepes (pH 7.5) for storage.

#### eEF-2K constructs (eEF-2K, C-LiNK, and C-LiNK_TR_)

A pET32a plasmid containing the designed C-LiNK sequence with an N-terminal tobacco etch virus (TEV) cleavage site and the stop codon before the C-terminal His-tag was purchased from GenScript. Recombinant eEF-2K constructs were expressed and purified as previously described, with minor adjustments to the purification protocol, as described below (32). For *in vitro* autophosphorylation studies, constructs were expressed in combination with λ-phosphatase (pCDF-duet vector). Cell lysis and Ni-NTA affinity chromatography were performed as previously described (32). Following the Ni-NTA elution, the protein concentration was estimated by Bradford assay (Bio-Rad), and the N-terminal His_6_ tag was cleaved by the addition of 1.5% (w/w) TEV protease and overnight dialysis into purification buffer C [20 mM Tris (pH 8), 150 mM NaCl, and 5 mM MgCl_2_]. The sample was then purified using the method previously described for the Mono Q anion exchange column (32), followed by further purification by gel-filtration chromatography (HiPrep 26/60 Sephacryl S-200 HR; Amersham Biosciences). The collected sample was then dialyzed into purification buffer D [25 mM Hepes (pH 7.5), 10% glycerol, 50 mM KCl, 0.1 mM EDTA, 0.1 mM EGTA, and 2 mM DTT] overnight and concentrated using an Amicon Ultra-15 Centrifugal Filter Unit (Millipore) for storage.

The C-LiNK_TR_ construct was designed by replacing the 359 to 489 segment of C-LiNK with a stretch of six glycine residues. The corresponding cDNA, codon-optimized for *E. Coli* expression, was synthesized commercially (GenScript) and inserted into a pET-24a vector (containing a stop codon before the C-terminal His tag). The C-LiNK_TR_ protein was coexpressed with λ-phosphatase (pCDF-duet vector) in BL21 (DE3) cells (New England BioLabs) and purified as previously described for eEF-2K_TR_ (18). The enzyme was further incubated with λ-phosphatase (∼150–1 molar ratio) both during dialysis (overnight at 4 °C) and before injection in the gel filtration column (room temperature, 2 h) in the presence of 1 mM MnCl_2_. The resulting enzyme lacked phosphorylation, as confirmed by electrospray ionization quadrupole time-of-flight MS.

#### Eukaryotic elongation factor 2

eEF-2 was purified from industrial yeast cake using a protocol similar to that of Jorgensen *et al.* (57). Briefly, yeast cells were suspended in purification buffer E [20 mM Hepes (pH 7.2), 10% glycerol, 1 mM PMSF, and 1 mM DTT] containing 300 mM KCl and mechanically lysed using a bead beater. The lysate was centrifuged at 17,400*g* for 25 min. The supernatant was clarified by ultracentrifugation at 50,000 RPM (70 Ti fixed-angle rotor) for 60 min. The supernatant was dialyzed to remove KCl and then centrifuged at 17,400*g* for 20 min. The resulting supernatant was filtered and applied to an SP FF 16/10 cation-exchange column. The sample was washed with 2 CV of purification buffer E containing 30 mM KCl and eluted over a 12 CV gradient up to 150 mM KCl. The eluted protein was applied to a Mono-Q column, washed with 2 CV of buffer E containing 40 mM KCl, and eluted over a 9 CV gradient up to 275 mM KCl. The collected fractions were reapplied to a Mono-Q column, washed with 2 CV of buffer D containing 40 mM KCl, and eluted over a 15 CV gradient up to 200 mM KCl. The protein was dialyzed into buffer F [25 mM Hepes (pH 7.5), 50 mM KCl, 2 mM DTT, and 5 mM MgCl_2_] overnight and concentrated using an Amicon Ultra-15 Centrifugal Filter Unit (Millipore) for storage.

### Multiangle light scattering

MALS experiments were performed as previously described (58). Briefly, experiments were performed at 25 °C on a DAWN HELEOS-II MALS photometer, equipped with an Optilab T-Rex refractive index detector and a Wyatt QELS dynamic light scattering detector. Samples were loaded onto a TSK-GEL G300PWXL size exclusion column (7.8 mm × 300 mm, with a pore size of 300 Å) using a Shimadzu LC-20AD HPLC system with buffer F as the solvent, at a flow rate of 0.4 ml/min. Molar mass (g/mol) was determined with 20 μl sample injections at a concentration of ∼20 μM. Astra 6 software (Wyatt Technology) was used to determine molar masses at the peak.

### Kinetics measurements and analysis

#### Manual mixing autophosphorylation assay

Enzyme (eEF-2K or C-LiNK) was incubated with or without CaM constructs (CaM, CaM_N_, or CaM_C_) in assay buffer G [25 mM Hepes (pH 7.5), 50 mM KCl, 10 mM MgCl_2_, 100 μM EGTA, 2 mM DTT, 20 μg/ml bovine serum albumin, 0.005% Brij-35] with 0, 1, or 50 μM free Ca^2+^ at 30 °C for 10 min before initiating the reaction with 1 mM Mg^2+^•ATP. The reaction was quenched at specified time points by addition into 2.3 volumes of hot SDS-loading buffer [50 mM Tris (pH 6.8), 1.6% SDS, 8% glycerol, 100 mM DTT, and 0.01% bromophenol blue] and further incubated at 95 °C for 5 min. Samples were analyzed for autophosphorylation by Western blotting using specific antibodies for eEF-2K (Santa Cruz Biotechnology) and its phosphorylated forms at *p*T348, *p*S445, or *p*S500 (ECM Biosciences). To correct for sample loss and loading error, the phospho-antibody signal for each sample was divided by its corresponding total eEF-2K signal. Data were converted to fraction phosphorylated and fit to Equation 1 for the apparent rate constant for autophosphorylation ($k_{\text{auto}}^{\text{app}})$.

(Eq. 1) $$Fraction\;phosphorylated=1-e^{-k_{\text{auto}}^{\text{app}}t}$$

#### Rapid quench autophosphorylation assay

T348 autophosphorylation reactions were initiated by rapidly mixing equal volumes of solution A (400 nM eEF-2K with 4 μM CaM or CaM_C_) and solution B (2 mM Mg^2+^•ATP) in assay buffer G with 150 μM CaCl_2_ (50 μM free Ca^2+^) at 30 °C on a KinTek RQF-3 apparatus. At specific time points, the reaction was quenched by the addition of buffer Q [200 mM KCl, 50 mM EDTA, and 10 mM EGTA in Hepes (pH 7.5)], then immediately dispensed into hot SDS-PAGE sample loading buffer, and incubated at 95 °C for an additional 5 min. Samples were analyzed for incorporation of phosphate at T348 by Western blotting. To correct for sample loss and loading error, the signal from the *p*T348 antibody for each sample was divided by its corresponding total eEF-2K signal. Data were converted to fraction phosphorylated and fit to Equation 1 for the apparent rate constant for autophosphorylation ($k_{\text{auto}}^{\text{app}})$.

#### General method for steady-state kinetic activity assays

eEF-2K or C-LiNK activity was assayed at 30 °C in assay buffer G. Reactions were initiated by adding 1 mm ATP with [γ-^32^P]-ATP (Revvity) at a specific activity of 100 to 1000 cpm/pmol and 1 mM MgCl_2_. The activity was determined by calculating the rate of phosphate incorporation into a substrate peptide or yeast eEF-2.

Incorporation of phosphate into a PepS, Ac-RKKYKFNEDTERRRFL-Amide (Peptide 2.0), was measured by removing 10 μl aliquots and spotting onto P81 ion exchange cellulose filter paper (Lab Alley Essential Chemicals) at 1 min intervals over 5 min. Spotted papers were immediately placed into 50 mM phosphoric acid. After completing the assay, all papers were washed three times in 50 mM phosphoric acid and once in acetone (each wash lasting 10 min), followed by air drying. The amount of radioactivity associated with each paper was determined by measuring counts per minute (cpm) values in 1 ml CytoScint Scintillation fluid (MP Biomedicals) on a Tri-Carb 2910/TR liquid scintillation analyzer (PerkinElmer). The rate of phosphate incorporation (nmol/sec) was divided by enzyme concentration (nmol) to obtain $k_{\text{obs}}$ (s^−1^) values.

Incorporation of phosphate into eEF-2 was measured by quenching reactions at 2 min with the addition of hot SDS-PAGE loading buffer followed by heating at 95 °C for an additional 5 min. Samples were then resolved by SDS-PAGE and stained using Coomassie Brilliant blue. Images were captured by exposing the gel to a phosphor screen for approximately 2 h before imaging the screen on an Amersham Typhoon RGB Biomolecular Imager. The gels were then dried, and eEF-2 bands were excised. The radioactivity associated with each eEF-2 band was measured using a Tri-Carb 2910 TR liquid scintillation analyzer. The rate of phosphate incorporation (nmol/sec) was divided by enzyme concentration (nmol) to obtain $k_{\text{obs}}$ (s^−1^) values.

#### CaM dependence assays

eEF-2K dose–response assays were performed in assay buffer G using several CaM (CaM or CaM_C_) concentrations in the presence or absence of 150 μM CaCl_2_ (50 μM free). Assays performed without CaCl_2_ contained an additional 0.9 mM EGTA (1 mM total). Reactions against eEF-2 (5 μM) or peptide (150 μM) were performed with 0.5 or 1 nM eEF-2K, respectively. Data were plotted as $k_{\text{obs}}$ against the concentration of CaM and fit to Equation 2 to obtain $k_{\text{obs}}^{\max}$ the maximal observed rate with saturating CaM under the specified conditions) and $K_{\text{CaM}}$ (the concentration of CaM required for half-maximal activity).

(Eq. 2) $$k_{\text{obs}}=k_{\text{obs}}^{\max}\frac{\left[E\right]+\left[\text{CaM}\right]+K_{\text{CaM}}-\sqrt{{\left(\left[E\right]+\left[\text{CaM}\right]+K_{\text{CaM}}\right)}^{2}-4\left(\left[E\right]\left[\text{CaM}\right]\right)}}{2\left[E\right]}$$

[E] is equal to the enzyme concentration in the assay, and [CaM] is the concentration of the CaM construct.

#### Substrate dependence assays

The activity of 1 nM eEF-2K or C-LiNK was measured in assay buffer G with 150 μM CaCl_2_ (50 μM free Ca^2+^) using several concentrations of PepS. Data were plotted as $k_{obs}$ against the concentration of PepS and fit to Equation 3 to obtain best-fit values for $k_{\text{cat}}^{\text{app}}$ and $K_{m}^{\text{app}}$.

(Eq. 3) $$k_{obs}=\frac{k_{\text{cat}}^{\text{app}}\left[\text{PepS}\right]}{K_{m}^{\text{app}}+\left[\text{PepS}\right]}$$

[PepS] is the PepS concentration.

#### Ca^2+^ dependence assays

The activity of 1 nM eEF-2K or C-LiNK was measured in assay buffer G with an additional 0.9 mM EGTA (1 mM total) and different concentrations of CaCl_2_. The concentration of free CaCl_2_ in the assay was calculated using the Ca-Mg-ATP-EGTA calculator v1.0 (59), and data were plotted as $k_{\text{obs}}$ for the calculated concentration of free CaCl_2_ ([Ca^2+^]_free_).

### Mass spectrometric methods

Bottom-up digestion with LC-MS/MS analysis was undertaken to identify the phosphorylation sites on C-LiNK. C-LiNK (50 μg) in 50 mM ammonium bicarbonate buffer (pH ∼8) was reduced with 5 mM DTT at 55 °C for 30 min, followed by alkylation with 15 mM iodoacetamide for 30 min in the dark before 5 mM DTT was added to stop alkylation. Trypsin was introduced at a 1:40 (w/w) protease:protein ratio, and the solution was incubated for 24 h at 37 °C. The resulting proteolyzed solution was purified with a C18 micro spin column and eluted using 70% acetonitrile. The samples were dried using a SpeedVac and reconstituted in a solution containing 2% acetonitrile with 0.1% formic acid for subsequent LC-MS/MS analysis.

The digest was analyzed using a Dionex RSLC 3000 nano-LC system (Thermo Fisher Scientific) interfaced to an Orbitrap mass spectrometer. Approximately 200 ng (1 μl injection volume) of the digested protein was injected into an in-house packed 3 cm C-18 trapping column (3 μm, 300 Å pore size, 75 μm ID). Peptides were eluted into an in-house packed 20 cm C-18 analytical column (1.8 μm, 300 Å pore size, 75 μm, New Objective). Separation was performed using mobile phase A consisting of water and mobile phase B consisting of acetonitrile (both containing 0.1% formic acid). Peptide separation was achieved over a 60-min linear gradient of mobile phase B (2% to 90%) at a flow rate of 300 nl/min. MS analysis was performed using an Eclipse Orbitrap mass spectrometer (Thermo Fisher Scientific Instruments), and the sample was analyzed in triplicate. MS1 spectra were collected with a resolution of 30,000 at an *m/z of 200*. MS/MS spectra were acquired using the same resolution with a 25% normalized collision energy applied for collisional activation. Bottom-up data processing and analyses were performed with Byonic software (Protein Metrics). A mass tolerance limit of 10 ppm was used to validate both precursor and fragments. Posterior error probability scores and delta scores were used to evaluate the confidence of the identified peptides.

### Crystallization of C-LiNK_TR_ and structure determination

Crystallization was performed using a 9.4 mg/ml protein stock solution in 20 mM Tris (pH 7.5), 100 mM NaCl, 1 mM tris(2-carboxyethyl)phosphine, 1 mM ADP, and 0.35 mM CaCl_2_. The diffraction data were collected at the NSLS-II 17-ID-1 beamline on a crystal obtained by vapor diffusion by mixing 2 μl of protein with 1 μl of a 18.8% w/v PEG3350 (Hampton Research) and 314 mM magnesium acetate (pH not adjusted) solution in a 24-well plate at room temperature. The crystallization conditions were found by optimizing a hit observed in well G1 of the NeXtal PEGs Suite (NeXtal Biotechnologies). The data were processed using autoPROC (60), and the structural model was generated using the Phaser and Refine modules within the Phenix suite (61) and Coot (62).

### Cell-based assays

#### Constructs

eEF-2K and C-LiNK coding sequences were subcloned into pcDNA3 using HindIII and XhoI restriction sites at the 5′ and 3′ ends, respectively, to generate the eEF-2K pcDNA3 and C-LiNK pcDNA3 expression constructs.

#### C-LiNK plasmid titration

A total of 0.15 × 10^6^ MCF10A *eef2k*^*−/−*^ cells (Sigma-Aldrich), maintained as described previously (without the use of penicillin or streptomycin) (19), were plated per well in a 35 mm plate. The following day, Lipofectamine 3000 (Invitrogen) was used to transfect cells with 0.1 μg of eEF-2K pcDNA3 or 0.1 μg, 0.5 μg, or 2 μg of C-LiNK pcDNA3 plasmid per well. All samples were transfected with a total of 2 μg of plasmid, with the difference between samples made up by adding empty pcDNA3 plasmid. Twenty-four hours later, the cells were washed twice with cold PBS and then lysed in M-PER lysis buffer supplemented with Halt protease and phosphatase inhibitor cocktail (Thermo Fisher Scientific). Lysates were cleared by centrifugation at 10,000*g* for 10 min, and the protein concentration in the lysate was analyzed using the Bradford assay (Bio-Rad). As described below, 30 μg of lysate was subjected to SDS-PAGE and Western blotting.

#### Cellular treatments

MCF10A *eef2k*^*−/−*^ cells were transfected as described above with either 0.5 μg empty pcDNA3, 0.1 μg eEF-2K pcDNA3 and 0.4 μg empty pcDNA3, or 0.5 μg C-LiNK pcDNA3. Transfected cells were treated with full media (NT) or starved the following day by replacing media with Dulbecco’s PBS (starved, STRV) for 2 h. Cells were then washed and lysed, and protein concentrations were determined as described above. Samples were subjected to Western blot analysis as described below, using 30 μg of lysate. Relative eEF-2 phosphorylation on Thr-56 was determined by normalization of *p*eEF-2 to total eEF-2 for each sample. Phosphorylated eEF-2 from the lysate of starved cells expressing eEF-2K (STRV) was normalized to 1, and *p*eEF-2 levels from all samples were quantified relative to STRV for each experiment.

### Protein detection by Western blotting

30 *μ*g of protein from cell lysates, as determined by the Bradford assay (Bio-Rad) or a specified amount of recombinantly purified protein was resolved by SDS-PAGE, and then transferred to PVDF membranes at 4 °C for 16 h at 30 V in transfer buffer [25 mM Tris, 192 mM glycine, and 20% v/v methanol]. Membranes were then probed for total eEF-2K (C-12, Santa Cruz Biotechnologies), *p*T348 eEF-2K (ECM Biosciences), *p*445 eEF-2K (ECM Biosciences), *p*S359 eEF-2K (Invitrogen), pS500 eEF-2K (ECM Biosciences), total eEF-2 (Santa Cruz Biotechnologies), *p*Thr-56 eEF-2 (Cell Signaling), or CaM (C-lobe epitope, Cell Signaling). Primary antibodies were detected by corresponding goat anti-rabbit or anti-mouse secondary antibodies (LI-COR).

Additional experimental procedures and protocols are available in the Supporting information.

## Data availability

The structure factors and coordinates have been deposited in the Protein Data Bank (PDB) with accession code 9OCW. All other data are available upon request to the corresponding authors.

## Supporting information

This article contains supporting information.

## Conflict of interest

The authors declare that they have no conflicts of interest with the contents of this article.
