# Supplementary material for: The critical role of the C-terminal lobe of calmodulin in activating eukaryotic elongation factor 2 kinase
Source: J Biol Chem. 2025 Aug 28;301(10):110650. doi: 10.1016/j.jbc.2025.110650 (PMC12509998; doi:10.1016/j.jbc.2025.110650)
Supplement: Supporting Figures and Tables [file mmc1.pdf]

SUPPORTING INFORMATION

for

The Critical Role of the C-terminal Lobe of Calmodulin in Activating Eukaryotic Elongation Factor 2 Kinase

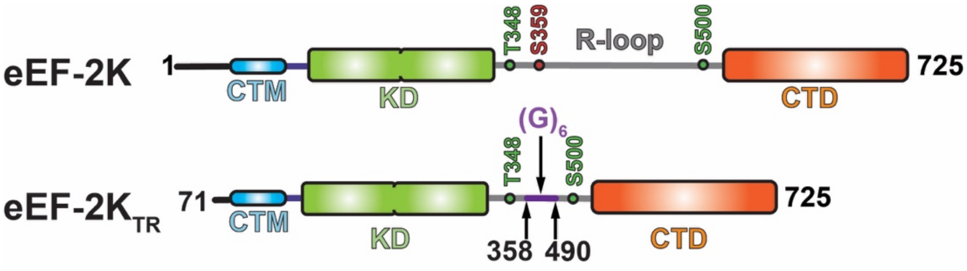

Fig. S1 Domain structure of eEF-2K. Full-length eEF-2K consists of 725 residues organized in an N-terminal calmodulin targeting motif (CTM), an α−kinase domain (KD), a regulatory loop (R-loop) containing multiple phosphorylation sites, and an all α−helical C-terminal domain (CTD). The stimulatory autophosphorylation sites, T348 and S500, and the inhibitory phosphorylation site, S359, discussed in the text, are indicated in green and red, respectively. The truncated eEF-2K construct (eEF-2K$_{TR}$) is missing 70 N-terminal residues, and 6 glycines have replaced the segment of the R-loop between residues 359 and 489.

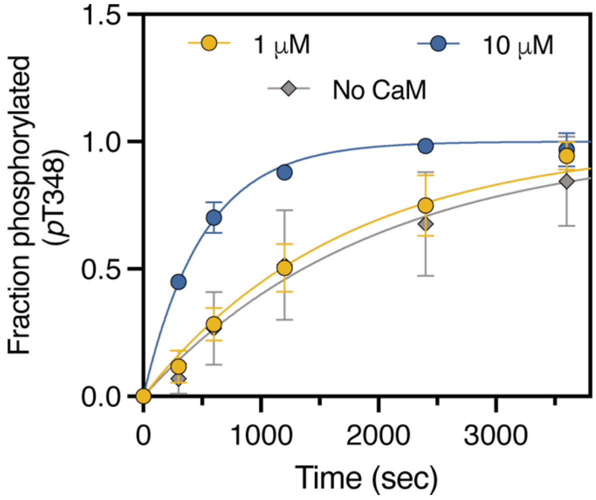

Fig. S2 Measurement of the ability of isolated CaM$_N$ to drive auto-phosphorylation on T348. 2 (CaM$_N$) or 3 (no CaM) independent experiments (depicted in Fig. 2A) were performed. Samples were quantified and plotted as the mean fraction of T348 phosphorylation ± standard deviation at each time point. Data were fit to Eq. 1 to determine best-fit k_{auto}^{app} values ± error for 1 μM CaM$_N$ (0.0006 ± 0.00004 s^{-1}), 10 μM CaM$_N$ (0.002 ± 0.00009 s^{-1}), and no CaM (0.0005 ± 0.00007 s^{-1}).

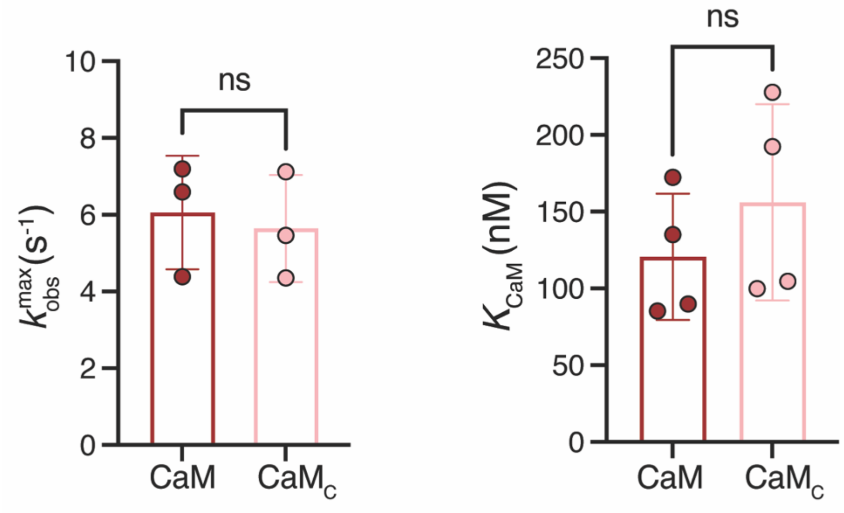

Fig. S3. Quantification of CaM dependence on eEF-2K activity against yeast eEF-2. Samples from Fig. 3B were excised from the gel and quantified by scintillation counting. Observed rate constants (k_{obs}) were plotted against CaM construct concentration, and each dataset was fitted to Eq. 2 to determine best-fit values for k_{obs}^{max} and K_{CaM}. Filled circles represent individual measurements; bars and vertical lines indicate mean ± standard deviation. The average k_{obs}^{max} values were 6.1 ± 1.5 s^{-1} for CaM and 5.6 ± 1.4 s^{-1} for CaM$_C$, while K_{CaM} values were 121 ± 41 nM and 156 ± 64 nM, respectively. Unpaired t-tests showed no significant difference between CaM and CaM$_C$ for k_{obs}^{max} (P = 0.74) or K_{CaM} (P = 0.39).

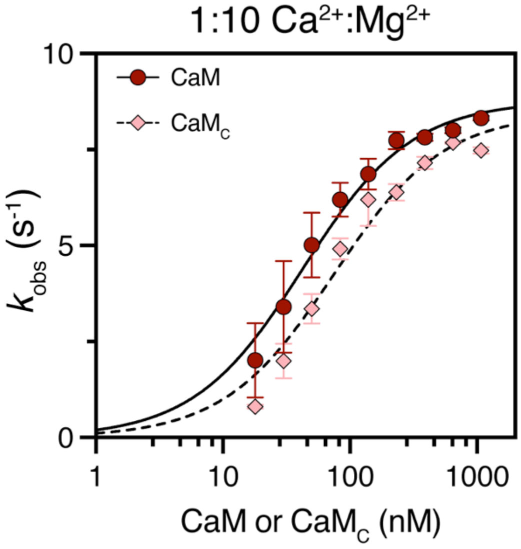

Fig. S4. Dependence of eEF-2K activity on CaM or CaM_C at a Ca^{2+}:Mg^{2+} ratio of 1:10. The activity of 1 nM eEF-2K against 150 µM PepS was measured using 1 mM [γ-^{32}P]-ATP in the presence of 1 mM free Ca^{2+} and 10 mM free Mg^{2+}. The dose-response of CaM or CaM_C was determined, with mean observed rate constants (k_{obs}) ± SD (n = 2) plotted against CaM construct concentration. Data were fitted to Eq. 2 to derive best-fit parameters: k_{obs}^{max} (CaM = 8.8 ± 0.3 s^{-1}; CaM_C = 8.5 ± 0.3 s^{-1}) and K_{CaM} (CaM = 41 ± 5 nM; CaM_C = 73 ± 9 nM). These K_{CaM} values closely match those previously reported at a Ca^{2+}:Mg^{2+} ratio of 1:200 (CaM = 67 ± 7 nM; CaM_C = 85 ± 10 nM), indicating no significant change in binding affinity.

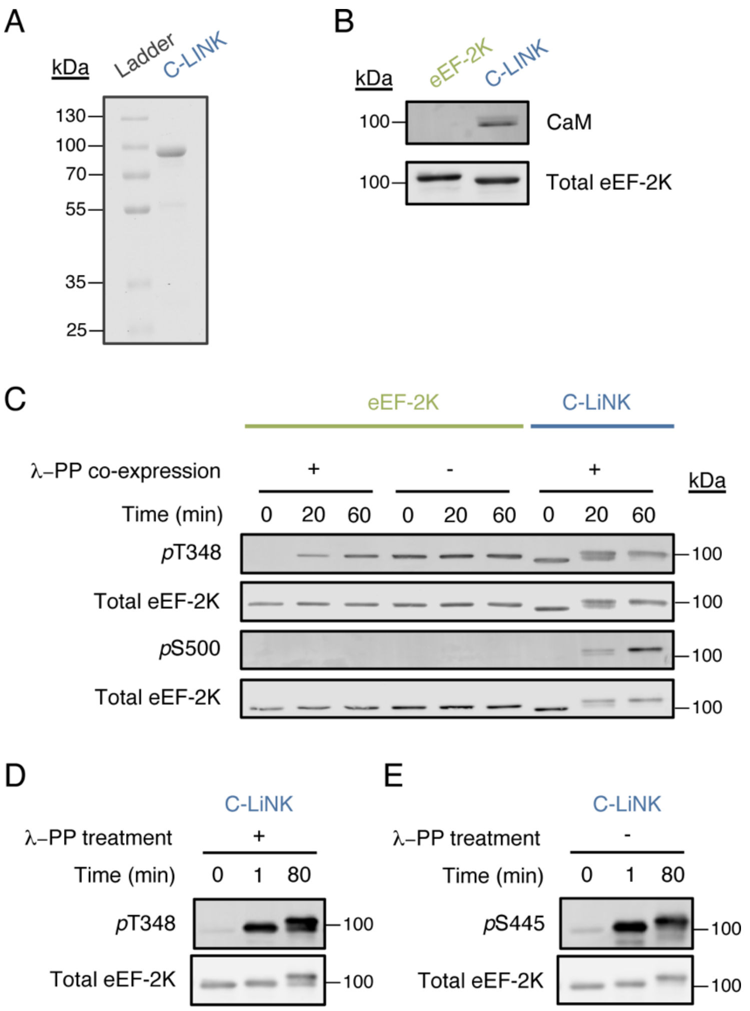

Fig. S5. Autophosphorylation of C-LiNK. (**A**) Purified C-LiNK (2 µg) was resolved on a 10% SDS-PAGE gel and visualized by Coomassie blue staining. (**B**) Western blot analysis of 0.5 µg eEF-2K or C-LiNK using antibodies specific for the C-terminus of eEF-2K (Santa Cruz, residues 436–725) and calmodulin (Cell Signaling, C-terminus). (**C**) Purified eEF-2K (± co-expression with λ-phosphatase) or C-LiNK (co-expressed with λ-phosphatase) were incubated with 1 mM ATP for 0, 30, or 60 minutes in the presence of 50 µM Ca^{2+}, then quenched with hot SDS-loading buffer. Western blots (0.125 µg protein loaded) probed total enzyme and autophosphorylation at T348 and S500. (**D–E**) C-LiNK co-expressed with λ-phosphatase was either treated or untreated with λ-phosphatase during purification (+/− λ-PP). Autophosphorylation assays were performed with 200 nM enzyme and 50 µM free $CaCl_2$. Reactions were initiated with 1 mM ATP and quenched at various time points with hot SDS loading buffer. Samples (150 ng enzyme) were analyzed by western blotting to detect (**D**) pT348, (E) pS445, and total eEF-2K.

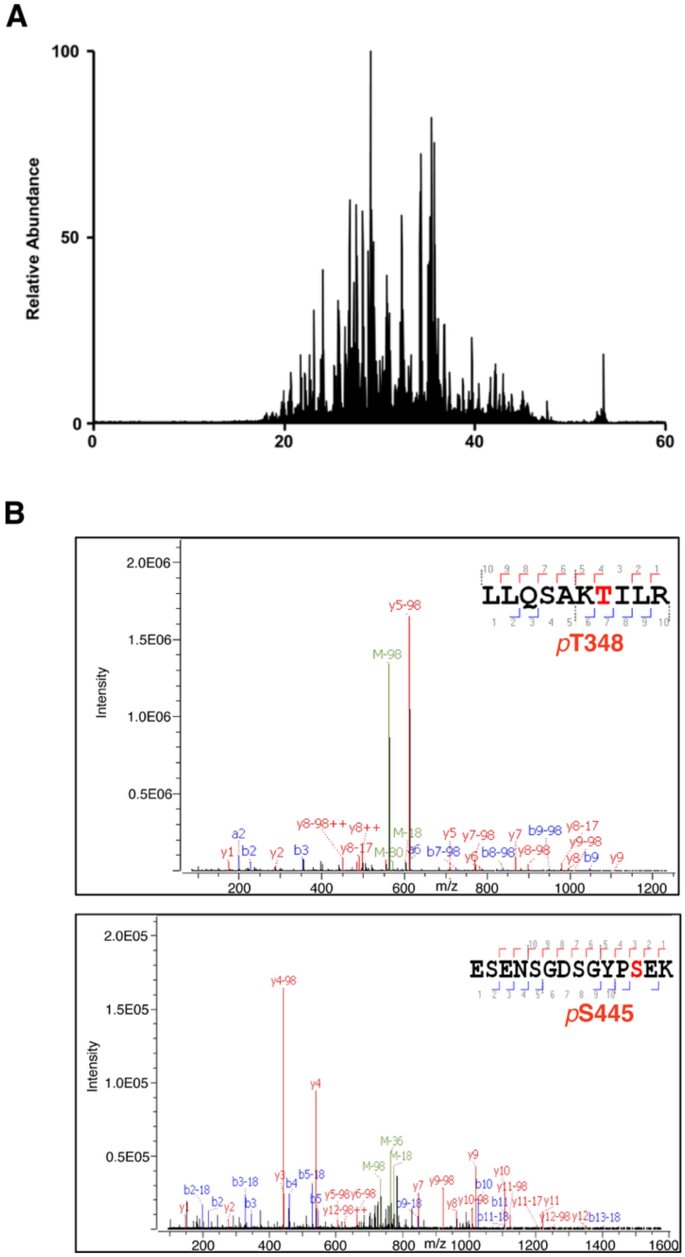

Fig. S6. Phosphorylation of expressed C-LiNK at T348. (A) Total ion chromatogram illustrating peptide separation during LC-MS/MS analysis. (**B**) CID fragmentation spectra of peptides confirming phosphorylation sites: the upper spectrum shows peptide 342-351 (LLQSAK*p*TILR, 2+) with phosphorylation at T348, which is highly abundant (>99%). The lower spectrum shows peptide 434-447 (ESENSGDSGYP*p*SEK, 2+) phosphorylated at S445, present at low abundance (~1%). Complete lists of assigned fragment ions are provided in Tables S1 and S2.

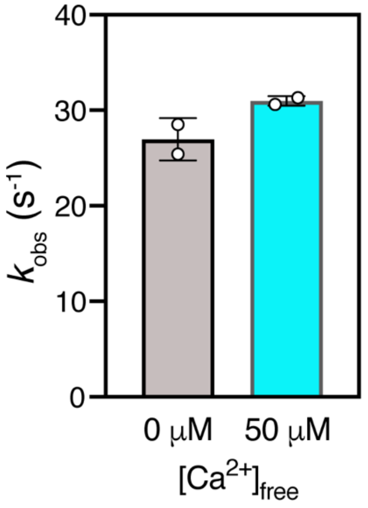

Fig. S7. Effect of Ca^{2+} on C-LiNK activity. C-LiNK (23 μM) was dialyzed for 19 hours against buffer containing 1 mM EGTA. The activity of 4 nM C-LiNK was then measured in assay buffer G containing 0.9 mM EGTA, using 150 μM PepS as substrate. $CaCl_2$ was added to reactions to achieve 50 μM free Ca^{2+}, while $MgCl_2$ was maintained at 10 mM free Mg^{2+} in all reactions. Reactions were initiated with 1 mM [γ-^{32}P]-ATP. Observed rate constants (k_{obs}) are shown as circles, with mean ± SD (n = 2) represented by filled bars and error bars, respectively.

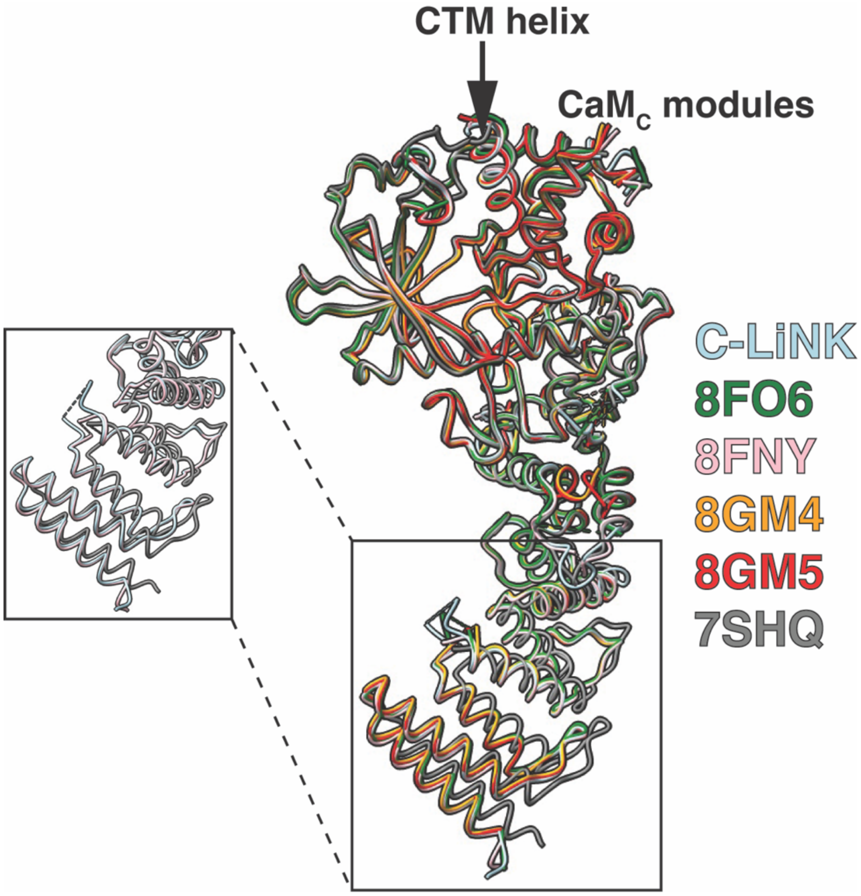

Fig. S8. C-LiNK$_{TR}$ adopts a similar overall conformation to the structural modules of the CaM•peEF-2K$_{TR}$ complex. Overlaying all available CaM•peEF-2K$_{TR}$ structures from the PDB with C-LiNK$_{TR}$ reveals that the overall conformations of the eEF-2K$_{TR}$ and CaM$_C$ modules are largely conserved. Notably, the 7SHQ structure displays a subtly different CTD conformation compared to other structures, including C-LiNK$_{TR}$ (the inset shows an expanded view highlighting the CTDs of C-LiNK$_{TR}$, 8FNY, and 7SHQ). This variation in the 7SHQ CTD likely reflects interdomain flexibility. This structure was solved using a crystal obtained under distinct crystallization conditions.

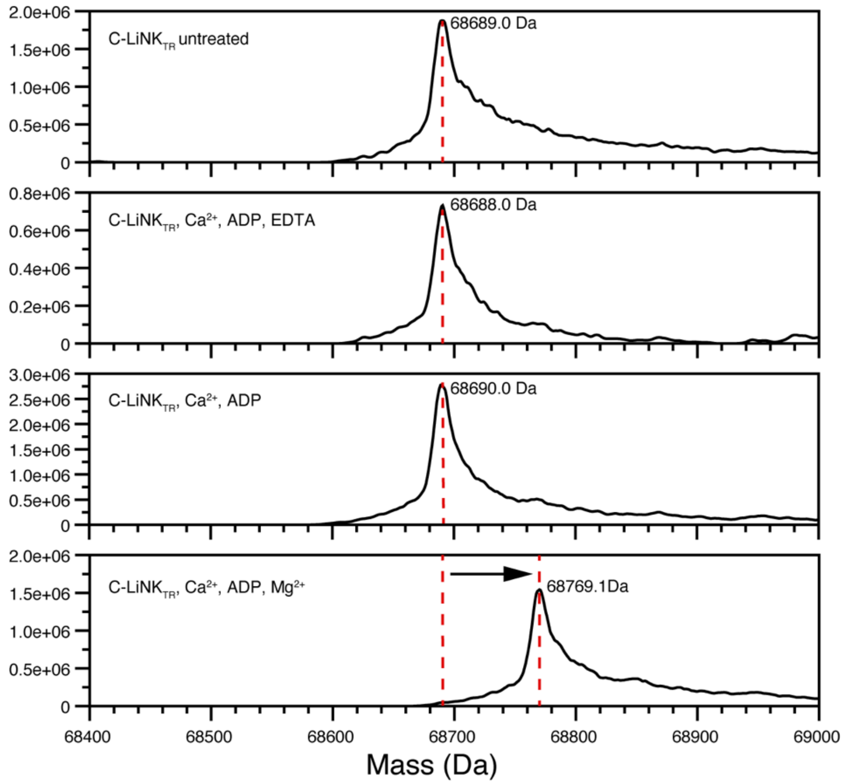

Fig. S9. Intact mass analysis of expressed C-LiNK$_{TR}$ by ESI-Q-TOF mass spectrometry. (Top panel) C-LiNK$_{TR}$ co-expressed with λ-phosphatase (λ-PP) and further treated with λ-PP as described in the main text. (Second panel) Protein stock (9.4 mg/mL) used for crystallography, prepared in buffer containing 20 mM Tris (pH 7.5), 100 mM NaCl, 1 mM TCEP, 1 mM ADP, and 0.35 mM $CaCl_2$, incubated with 20 mM EDTA. (Third panel) Protein stock used for crystallography without EDTA treatment. (Bottom panel) Protein stock incubated with 10 mM $MgCl_2$ for 24 hours at 4 °C. This sample shows a mass shift consistent with the addition of a single phosphate following Mg^{2+} incubation.

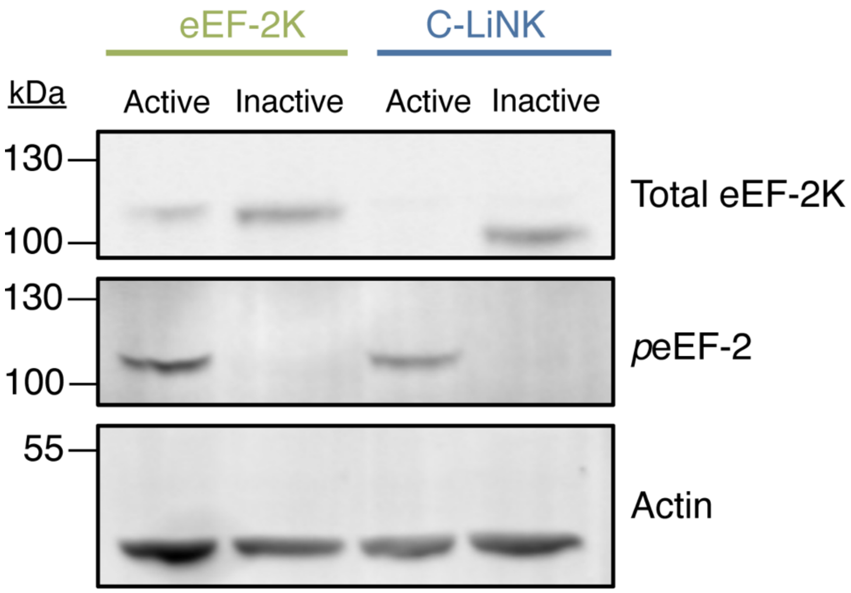

Fig. S10. Generation of a catalytically compromised C-LiNK variant restores protein levels. MCF10A *eef2k*$^{-}$/$^{-}$ cells were transiently transfected with 0.1 µg of plasmid encoding either eEF2K or catalytically inactive eEF2K (D284A mutant), both expressed from the pcDNA3 FLAG HA vector (Addgene #10792), or active C-LiNK and catalytically inactive C-LiNK (D290A mutant, where D290 in C-LiNK corresponds to D284 in eEF2K), both expressed from pcDNA3. After 16 hours, cells were lysed, and 40 µg of total protein lysate was analyzed by western blot using antibodies against eEF2K, phosphorylated eEF2 (p-eEF2), and actin as a loading control.

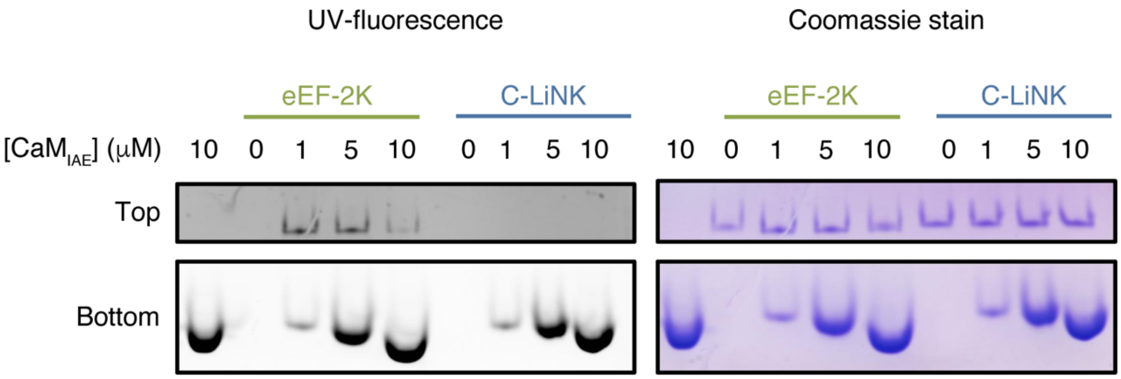

Fig. S11 Exogenous CaM does not bind C-LiNK. 400 nM eEF-2K was incubated with the indicated concentrations of CaM labeled with the fluorescent dye IAEDANS (CaM$_{IAE}$; the dye was covalently attached to position 75 in an R75C mutant of CaM) and 100 μM free $CaCl_2$ for 20 min at room temperature before running samples on a gradient (4-15%) native gel. Fluorescence was detected by exposing the gel to ultraviolet light for 0.5 (bottom) or 34.5 (top) seconds before capturing the image. The gel was Coomassie-stained to detect total protein.

Additional experimental procedures

Effects of increased Ca^{2+} to Mg^{2+} ratio on eEF-2K activity

1 nM eEF-2K activity was measured in buffer G (see main text) with 1.15 mM $CaCl_2$ (1 mM free Ca^{2+}) with varied CaM or CaM_C. Data were fit to Eq. 1.

Dephosphorylation of C-LiNK

To further dephosphorylate C-LiNK protein co-expressed with a plasmid containing λ-PP, it was treated with λ-PP during purification. The cleared cell lysate was incubated with Ni-NTA beads (Qiagen) for 1 hour at 4 ºC. In a chromatography column, the beads were washed with purification buffer containing 25 mM HEPES (pH 8), 100 mM NaCl, 20 mM Imidazole, and 0.03% Brij-30 (v/v), 1% 2-mercaptoethanol (v/v), 0.1 mM TPCK, 0.1 mM PMSF, and 1 mM benzamidine. The tagged C-LiNK was then eluted in 30 mL of a buffer containing 25 mM HEPES (pH 8), 250 mM Imidazole, and 0.03% Brij-30 (v/v), 1% 2-mercaptoethanol (v/v), 0.1 mM TPCK, 0.1 mM PMSF, and 1 mM benzamidine. λ-PP (New England Biolabs) was added to the eluted protein at a 1:250 ratio (λ-PP:C-LiNK), and 1 mM $MnCl_2$ was added. The sample was dialyzed against purification buffer with 1 mM $MnCl_2$ for 16 hours at 4 ºC. Following dialysis, the sample was re-purified through the Ni-NTA column before treating with TEV protease and continuing successive chromatographic steps.

Autophosphorylation of C-LiNK

C-LiNK was expressed in the presence of λ-PP. Further treatment of the enzyme with λ - PP is discussed above. The autophosphorylation reaction was performed in assay buffer G at 30 °C with 200 nM enzyme in the presence of 50 μM free $CaCl_2$. The reaction was initiated with 1 mM ATP, then quenched at various time points by adding hot SDS loading buffer. 150 ng of phosphorylated enzyme was run on SDS-PAGE, and phosphate incorporation was detected by western blotting for *p*T348 (ECM Biosciences) or *p*S445 (ECM Biosciences).

Effect of extended Ca^{2+} chelation on C-LiNK activity

Approximately 23 μM C-LiNK in purification buffer D (see main text) was dialyzed for 19 hours against assay buffer G with an additional 0.9 mM EGTA, for a total 1 mM EGTA. Subsequently, 4 nM dialyzed C-LiNK activity was measured against 150 μM PepS in assay buffer G, with or without $CaCl_2$ (50 μM free Ca^{2+}) and added $MgCl_2$ to achieve 10 mM free Mg^{2+} in solution. Reactions were initiated with 1 mM [γ- ^{32}P]-ATP, and time points were taken over 5 min.

Native protein detection

400 nM eEF-2K was incubated with indicated concentrations of IAEDANS-labeled CaM_{R75C} (CaM_{IAE}) in native buffer containing 5 mM HEPES (pH 6.8), 50 mM KCl, 100 μM EGTA, 150 μM $CaCl_2$, and 0.005% Brij for 20 min at room temperature before running samples on a gradient (4-15%) Mini-Protean TGX native gel (BioRad) using Tris/glycine native running buffer (pH 8.3; BioRad).

Table S1. Fragment ion assignments for phosphorylated peptide LLQSAK**T**ILR (*m/z* 611.85, 2+, retention time: 26.44 min) identified by LC-MS/MS-HCD following tryptic digestion of C-LiNK. Theoretical and observed masses of b- and y-ions are listed along with the corresponding mass differences and errors (ppm)

Ion Type	Number of residues	Theoretical Mass (Da)	Observed Mass (Da)	Mass difference (Da)	Mass Error (ppm)
a	2	199.1805	199.1799	-0.0006	-3.0
b	2	227.1754	227.1749	-0.0005	-2.2
b	9	1048.5802	1048.5698	-0.0104	-9.9
y	1	175.119	175.1186	-0.0004	-2.3
y	6	781.4332	781.4294	-0.0038	-4.9
y	7	868.4652	868.4613	-0.0039	-4.5
y	8	996.5238	996.5217	-0.0021	-2.1
y (2+)	8	498.7655	498.7642	-0.0013	-2.6
y (2+)	9	555.3075	555.306	-0.0015	-2.7

Number of residues indicates the number of amino acids contained in the fragment ion. For a- and b-type ions, this number is anchored relative to the N-terminus. For y-type ions, this number is anchored relative to the C-terminus. All ions are singly charged except for two doubly charged y-type ions.

Table S2. Fragment ion assignments for phosphorylated peptide ESENSGDSGYP**S**EK (*m/z* 783.28, 2+, retention time: 18.54 min) identified by LC-MS/MS-HCD following tryptic digestion of C-LiNK. Theoretical and observed masses of b- and y-ions are listed along with the corresponding mass differences and errors (ppm).

Ion Type	Number of residues	Theoretical Mass (Da)	Observed Mass (Da)	Mass difference (Da)	Mass Error (ppm)
b	2	217.0819	217.0812	-0.0007	-3.2
b	3	346.1245	346.123	-0.0015	-4.3
b	5	547.1994	547.1974	-0.002	-3.7
b	10	1026.3647	1026.3605	-0.0042	-4.1
y	1	147.1128	147.1123	-0.0005	-3.4
y	2	276.1554	276.1545	-0.0009	-3.3
y	4	540.2065	540.2045	-0.002	-3.7
y	7	847.3233	847.3195	-0.0038	-4.5
y	8	962.3503	962.347	-0.0033	-3.4
y	9	1019.3717	1019.3669	-0.0048	-4.7
y	10	1106.4038	1106.3987	-0.0051	-4.6
y	11	1220.4467	1220.4431	-0.0036	-2.9
y	12	1349.4893	1349.4889	-0.0004	-0.3

Number of residues indicates the number of amino acids contained in the fragment ion. For a- and b-type ions, this number is anchored relative to the N-terminus. For y-type ions, this number is anchored relative to the C-terminus.
